# The effect of *Helicobacter pylori* eradication on prognosis of postoperative early gastric cancer: a multicenter study

**DOI:** 10.1186/s12957-021-02343-x

**Published:** 2021-09-21

**Authors:** Liang Wang, Jinfeng Wang, Sha Li, Fei Bai, Hailong Xie, Hanguo Shan, Zhuo Liu, Tiexiang Ma, Xiayu Tang, Haibing Tang, Ang Qin, Sanlin Lei, Chaohui Zuo

**Affiliations:** 1 Graduates School, University of South China, 28 Zhengxiang Road, Hengyang, Hunan Province 421001 People’s Republic of China; 2 Department of Gastroduodenal and Pancreatic Surgery, Translational Medicine Research Center of Liver Cancer, Laboratory of Digestive Oncology, Hunan Cancer Hospital (Hunan Cancer Institute) & the Affiliated Cancer Hospital of Xiangya School of Medicine, Hunan Province Key Laboratory of Virology (Tumor Immunity), Central South University, 283 Tongzipo Road, Changsha, Hunan Province 410013 People’s Republic of China; 3 Cancer Research Institute, Department of Gastrointestinal Surgery of Second Affiliated Hospital, University of South China, 28 Zhengxiang Road, Hengyang, Hunan Province 421001 People’s Republic of China; 4 The Third Department of General Surgery, The Central Hospital of Xiangtan City, 120 Heping Road, Xiangtan City, Hunan Province 411100 People’s Republic of China; 5 Department of General Surgery, Yongzhou Central Hospital, Xiaoshui Road, Yongzhou City, Hunan Province 425000 People’s Republic of China; 6 Department of General Surgery, People Hospital of Qiyang County, Xiaoshui Road, Qiyang County, Hunan Province 426100 People’s Republic of China; 7 Department of General Surgery, The Second Xiangya Hospital of Central South University, 139 Renmin Middle Road, Changsha, Hunan Province 410011 People’s Republic of China

**Keywords:** Early gastric cancer (EGC), *Helicobacter pylori* (*H. pylori*), Prognosis, Risk factors

## Abstract

**Objective:**

To investigate the effect of *Helicobacter pylori* (*H. pylori*) eradication on the prognosis of postoperative early gastric cancer (EGC).

**Methods:**

This is a retrospective study based on data from 6 hospitals. We identified 429 patients with EGC who underwent curative gastrectomy from January 2010 to December 2016. All of the patients were tested for *H. pylori*. Patients were divided into two groups, the successful *H. pylori* eradication group (group A, 268 patients) and the non-*H. pylori* eradication group (group B, 161 patients), for calculating the disease-free survival (DFS) and overall survival (OS) of each group.

**Result:**

Positive node metastasis (hazard ratio (HR), 3.13; 95% confidence interval (CI), 1.84–5.32; *P* < 0.001), undifferentiated type (HR, 2.54; 95% CI, 1.51–4.28; *P* < 0.001), and non-*H. pylori* eradication (HR, 1.73; 95% CI, 1.08–2.77; *P* = 0.023) were statistically significantly independent risk factors of recurrence. Patient’s age ≥60 years old (HR, 3.32; 95% CI, 2.00–5.53; *P* < 0.001), positive node metastasis (HR, 3.71; 95% CI, 2.25–6.12; *P* < 0.001), undifferentiated type (HR, 3.06; 95% CI, 1.79–5.23; *P* < 0.001), and non-*H. pylori* eradication (HR, 1.83; 95% CI, 1.11–3.02; *P* = 0.018) were statistically significantly independent risk factors of overall survival.

**Conclusion:**

*H. pylori* eradication treatment could prevent the recurrence of postoperative EGC to prolong the overall survival of patients with EGC.

## Introduction

Gastric cancer (GC) is the fifth most common cancer and the third deadly cancer, with more than 780,000 annual deaths all over the world in 2018 [1]. The highest age-standardized incidence is found in Eastern Asian populations, and the lowest in Northern Africa populations. The age-standardized incidence in South Korea is 32.1 and 13.2 per 100,000 individuals in men and women, respectively. *Helicobacter pylori* (*H. pylori*) plays a vital role in the occurrence and development of GC. Strong epidemiological and clinical evidence has shown that *H. pylori* infection is positively correlated with active chronic gastritis, peptic ulcers, atrophic gastritis, intestinal metaplasia, and GC [2, 3]. People who were infected with *H. pylori* have a more than threefold increase in risk of GC [4]. The 5-year survival rate for GC in 2005–2009 was 54–58% in Japan and South Korea [5], while the 5-year survival rate of EGC is more than 90%. A previous meta-analysis has suggested that *H. pylori* eradication reduces the risk of GC [6]. However, the role of *H. pylori* eradication on the effect of prognosis on EGC has not been thoroughly examined. Recurrence is still a key factor affecting the survival of patients with EGC. This study aims to investigate the effect of *H. pylori* eradication on the postoperative prognosis of patients with EGC.

## Methods

### Study population

We identified 429 patients with EGC who underwent curative gastrectomy for GC from January 2010 to December 2016 from six hospitals (253 patients from Hunan Province Cancer Hospital, 109 patients from the Second Xiangya Hospital of Central South University, 23 patients from the Second Affiliated Hospital of University of South China, 19 patients from the Central Hospital of Xiangtan City, 17 patients from Yongzhou Central Hospital, and 8 patients from People Hospital of Qiyang County). All patients were tested for *H. pylori* by a carbon-14 (C-14) breath test. All cases were pathologically diagnosed as EGC and were consulted by the Multiple Disciplinary Team (MDT) in each center. Curative gastrectomy was performed on all patients who did not undergo or did not wish to undergo neoadjuvant chemotherapy. For a long time, there is no international consensus on whether patients with GC can benefit from *H. pylori* eradication treatment after surgery. In clinical practice, whether or not to treat with *H. pylori* eradication is mainly up to the patient’s choice. The exclusion criteria are negative *H. pylori* and *H. pylori* eradication failure. Finally, a total of 429 patients included were divided into two groups, the successful *H. pylori* eradication group (group A) and the non-*H. pylori* eradication group (group B). Group A received standard triple therapy (esomeprazole 20 mg bis in die (bid), amoxicillin 1 g bid, and clarithromycin 0.5 g bid) orally for 7 days. *H. pylori* were identified by a C-14 breath test 1 month after treatment.

### Definition

EGC is defined as gastric adenocarcinoma limited to the mucosa or submucosa, no matter whether it has lymph node metastasis or not. According to the Japanese Gastric Cancer Association principles [7], the stomach is anatomically divided into three portions, the lower part, the middle part, and the upper part. The tumor size is measured by the maximum diameter of the tumor. The macroscopic classification of GC was divided into three types, elevated type (types 0-I, 0-I + II a, 0-II a, 0-II a + II b, 0-II a + II c), flat type (type 0-II b), and depressed type (types 0-II c, 0-III, 0-II c + II a, and 0-III + II a). According to WHO criteria, the histological classification of GC was classified into two different types, undifferentiated type (including mucinous adenocarcinoma, poorly differentiated tubular adenocarcinoma, and signet-ring cell carcinoma) and differentiated type (including well and moderately differentiated tubular adenocarcinoma and papillary adenocarcinoma).

### Follow-up

Patients with EGC were followed up regularly after the radical gastrectomy. The last follow-up date was July 30, 2020. The patients were followed up every 6 months for the first 3 years after surgery and then once a year until death or loss to follow-up. The follow-up information, including the time of patient relapse or death, was collected from hospital information systems, patients, or patients’ family members. Overall survival (OS) was calculated from the date of pathological diagnosis to death or the last date of follow-up. Disease-free survival (DFS) was calculated from the date of pathological diagnosis to recurrence or the last date of follow-up.

### Statistical analysis

The data were statistically processed using SPSS22.0 statistical software. Student *t*-test was used for comparing numerical data and the Pearson chi-square test for comparing categorical data. Data are shown as the mean ± standard deviation. Univariate and multivariate analysis were performed by Cox proportional hazards regression model. The survival curve was traced using the Kaplan–Meier method, the difference between curves was tested using the log-rank test. A 2-tailed *P* value less than 0.05 was considered statistically significant difference.

## Result

### Patients’ characteristic

The baseline characteristics of these 429 patients are shown in Table 1. Group A and group B have no significant differences in sex ratio, age, tumor size, the depth of invasion, histological classification, and macroscopic classification. The average age of patients was 56.01 ± 9.53 years. The percentage of female patients (36.6%, 157/429) was lower than male patients (63.4%, 272/429) among patients with EGC. The average size of the tumor was 24.5 ± 10.6 mm. The most common tumor location was the lower third of the stomach (53.8%, 231/429), followed by the middle (32.6%, 140/429) and upper (13.5%, 58/429) parts of the stomach. The most common gross appearance was the depressed type (53.4%, 229/429), followed by the flat type (31.7%, 136/429) and elevated type (14.9%, 64/429). The percentage of differentiated type (49.2%, 211/429) is very similar to undifferentiated type (50.8%, 218/429). Totally, 210 (49.0%) patients had submucosal invasion and 41 patients (16.6%) had lymph node metastasis.

**Table 1:** Baseline characteristics of patients with EGC

	***Total***	**Group A**	**Group B**	***P value***
Patient number	429	268	161	
Age (mean ± SD), years	55.94 ± 9.50	55.90 ± 9.49	56.01 ± 9.53	0.908
≥60	157 (36.6%)	99 (36.9%)	58 (36.0%)	0.849
< 60	272 (63.4%)	169 (63.1%)	103 (64.0%)	
Gender				0.667
Male	272 (63.4%)	172 (64.2%)	100 (62.1%)	
Female	157 (36.6%)	96 (35.8%)	61 (38.9%)	
Tumor location				0.990
Upper	58 (13.6%)	36 (13.4%)	22 (13.7%)	
Middle	140 (32.6%)	87 (32.5%)	53 (32.9%)	
Lower	231 (53.8%)	145 (54.1%)	86 (53.4%)	
Tumor size, mean ± SD, mm	24.5 ± 10.6	23.9 ± 10.5	25.3 ± 10.7	0.176
≥20	267 (62.2%)	159 (59.3%)	108 (67.1%)	0.109
< 20	162 (37.8%)	109 (40.7%)	53 (32.9%)	
Depth of invasion				0.525
Mucosa	219 (51.0%)	140 (52.2%)	79 (49.1%)	
Submucosa	210 (49.0%)	128 (47.8%)	82 (50.9%)	
Macroscopic type				0.510
Depressed	229 (53.4%)	147 (54.9%)	82 (50.9%)	
Flat	136 (31.7%)	85 (31.7%)	51 (31.7%)	
Elevate	64 (14.9%)	36 (13.4%)	28 (17.4%)	
Node metastasis				0.924
Positive	71 (16.6%)	44 (16.4%)	27 (16.8%)	
Negative	388 (83.4%)	224 (83.6%)	134 (83.2%)	
Histology				0.404
Undifferentiated type	218 (50.8%)	132 (49.3%)	86 (53.4%)	
Differentiated type	211 (49.2%)	136 (50.7%)	75 (46.6%)	

The data are expressed as the number, with the percentage in parentheses

*SD* Standard deviation

### Risk factors of recurrence among postoperative with EGC

We conducted univariate Cox analysis to identify the prognostic significance of clinicopathological factors for DFS. Positive node metastasis (HR, 3.17; 95% CI, 1.92–5.21; *P* < 0.001), submucosal invasion (HR, 1.84; 95% CI, 1.15–2.94; *P* = 0.011), non-*H. pylori* eradication (HR, 1.64; 95% CI, 1.03–2.61; *P* = 0.037), and undifferentiated type (HR, 2.90; 95% CI, 1.75–4.79; *P* < 0.001) were found to be risk factors for recurrence in patients with EGC. Further adjustment of covariate factors using multivariate Cox analysis identified positive node metastasis (HR, 3.13; 95% CI, 1.84–5.32; *P* < 0.001), undifferentiated type (HR, 2.54; 95% CI, 1.51–4.28; *P* < 0.001), and non-*H. pylori* eradication (HR, 1.73; 95% CI, 1.08–2.77; *P* = 0.023) as statistically significantly independent risk factors of recurrence shown in Table 2.

**Table 2:** Predictive factors of recurrence of postoperative EGC

	**Univariate**			**Multivariate**		
	**HR**	**95% CI**	***P* value**	**HR**	**95% CI**	***P* value**
Age (≥60 vs < 60)	1.41	0.88–2.25	0.159	1.32	0.81–2.15	0.266
Gender (female vs male)	0.89	0.55–1.44	0.648	1.19	0.73–1.96	0.489
Tumor location (upper vs others)	1.06	0.54–2.07	0.864	1.02	0.52–2.01	0.960
Tumor size (≥2 cm vs < 2 cm)	1.00	0.63–1.61	0.987	0.96	0.59–1.54	0.850
Depth of invasion (submucosa vs mucosa)	1.84	1.15–2.94	0.011	2.26	1.39–3.67	0.001
Macroscopic type (depressed vs others)	1.24	0.78–1.98	0.356	1.36	0.85–2.18	0.195
Node metastasis (yes vs no)	3.17	1.92–5.21	< 0.001	3.13	1.84–5.32	< 0.001
Histology (undifferentiated vs differentiated type)	2.90	1.75–4.79	< 0.001	2.54	1.51–4.28	< 0.001
*H. pylori* eradication (no vs yes)	1.64	1.03–2.61	0.037	1.73	1.08–2.77	0.023

*H. pylori Helicobacter pylori*, *HR* Hazard ratio, *CI* Confidence interval

### Risk factors of overall survival among postoperative with EGC

We also conducted univariate Cox analysis to identify the prognostic significance of clinicopathological factors for OS. Patient’s age ≥60 years old (HR, 3.40; 95% CI, 2.08–5.55; *P* < 0.001), positive node metastasis (HR, 3.71; 95% CI, 2.25–6.12; *P* < 0.001), submucosal invasion (HR, 1.86; 95% CI, 1.14–3.04; *P* = 0.013), tumor located in the upper third part (HR, 2.02; 95% CI, 1.13–3.61; *P* = 0.017), non-*H. pylori* eradication (HR, 1.68; 95% CI, 1.03–2.74; *P* = 0.036), and undifferentiated type (HR, 3.06; 95% CI, 1.79–5.23; *P* < 0.001) were risk factors for overall survival in patients with EGC. In multivariate analysis, patient’s age ≥60 years old (HR, 3.32; 95% CI, 2.00–5.53; *P* < 0.001), positive node metastasis (HR, 3.71; 95% CI, 2.25–6.12; *P* < 0.001), undifferentiated type (HR, 3.06; 95% CI, 1.79–5.23; *P* < 0.001), and non-*H. pylori* eradication (HR, 1.83; 95% CI, 1.11–3.02; *P* = 0.018) were found to be statistically significant independent risk factors of overall survival shown in Table 3.

**Table 3:** Predictive factors of overall survival of postoperative EGC

	**Univariate**			**Multivariate**		
	**HR**	**95%CI**	***P* value**	**HR**	**95%CI**	***P* value**
Age (≥60 vs < 60)	3.40	2.08–5.55	< 0.001	3.32	2.00–5.53	< 0.001
Gender (female vs male)	0.90	0.55–1.49	0.689	1.43	0.84–2.44	0.186
Tumor location (upper vs others)	2.02	1.13–3.61	0.017	1.68	0.92–3.07	0.091
Tumor size (≥2 cm vs < 2 cm)	1.23	0.74–2.03	0.427	1.22	0.73–2.05	0.456
Depth of invasion (submucosa vs mucosa)	1.86	1.14–3.04	0.013	2.13	1.28–3.54	0.004
Macroscopic type (depressed vs others)	0.87	0.61–1.24	0.450	1.45	0.88–2.39	0.146
Node metastasis (yes vs no)	3.71	2.25–6.12	< 0.001	3.57	2.05–6.22	< 0.001
Histology (undifferentiated vs differentiated type)	3.06	1.79–5.23	< 0.001	2.12	1.20–3.72	0.009
*H. pylori* eradication (no vs yes)	1.68	1.03–2.74	0.036	1.83	1.11–3.02	0.018

*H. pylori Helicobacter pylori*, *HR* Hazard ratio, *CI* Confidence interval

### Prognosis and survival analysis

**Fig. 1:**
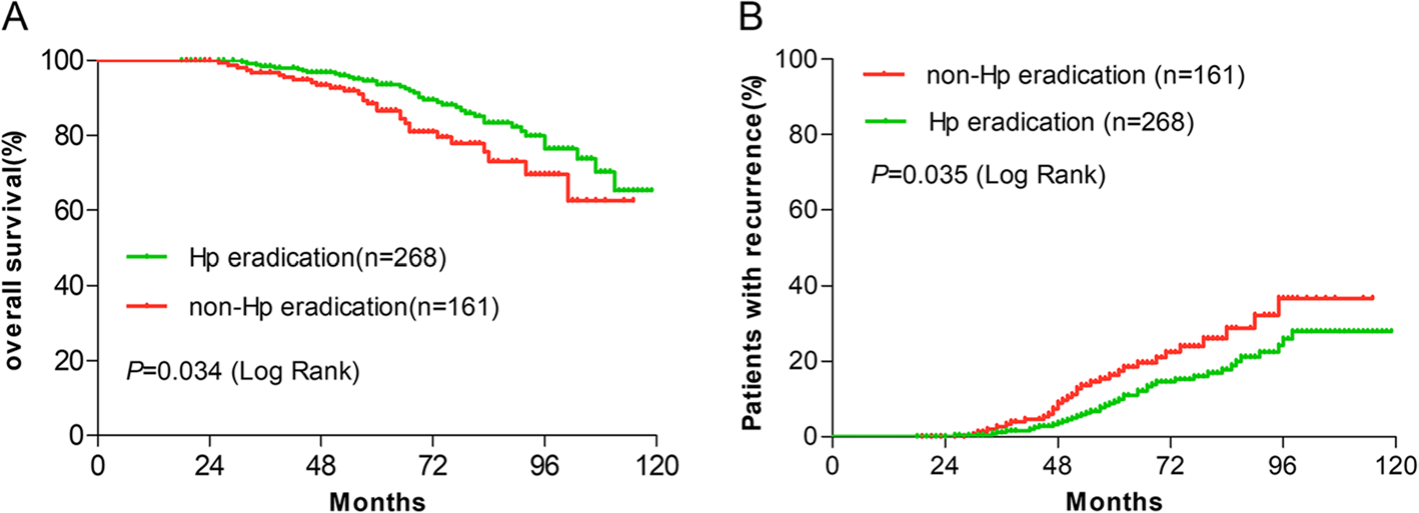
Kaplan–Meier analysis of the rate of overall survival and recurrence. As shown in Fig. 1, during a median follow-up of 69 months, 38 of 268 (14.2%) patients died in group A and 29 of 161 (18.0%) patients died in group B (**A**). There were 38 of 268 (15.3%) patients with recurrence in group A and 29 of 161 (19.9%) patients with recurrence in group B (**B**)

Sixty-seven patients (15.6%) died during a median follow-up of 69 months (range from 18 to 119 months). Three- and 5-year survival rates were separately 98.5% and 93.6% in group A and separately 96.9% and 86.6% in group B (Fig. 1A). It is obvious that the survival rate of group A was higher than that of group B, and the difference was statistically significant (log-rank *P* = 0.034). There were 73 patients (17.0%) with recurrence during the follow-up, 3- and 5-year recurrence rates were separately 1.2% and 9.3% in group A, and separately 2.6% and 16.4% in group B (Fig. 1B). It is shown that the recurrence rate was lower in group A than in group B (log-rank *P* = 0.035).

## Discussion

The occurrence of GC is a long-term process involving multiple factors. The underlying causes of GC are not fully understood. Since Warren and Mashall discovered *H. pylori* in 1983, the WHO concluded in 1994 that *H. pylori* is a carcinogen and plays a causative role in the pathogenesis of GC [8]. *H. pylori*-induced chronic inflammation and cancerization are associated with interaction of bacteria, host, and environment which forms a complex network to respond to inflammation and promote damage repair. *H. pylori* eradication, used to treat functional dyspepsia and prevent peptic ulcer disease that relapses after early ulcers, was found to prevent GC in a meta-analysis, showing a 50% reduction in risk [9, 10]. Hwang et al. found that *H. pylori* eradication may prevent intestinal-type GC by regression of atrophic gastritis and intestinal metaplasia in a prospective study for up to 10 years [11].

Although the mechanism of *H. pylori* causing GC has been extensively studied, few studies have been conducted on whether *H. pylori* eradication after the occurrence of GC can prevent the recurrence of GC and its effect on the prognosis after radical gastrectomy. For a long time, there is no international consensus on whether patients with GC can benefit from *H. pylori* eradication treatment. In this study, we found that the tumor located in the upper third was a risk factor for the overall survival of patients with EGC in univariate analysis, but not in multivariate analysis, and Xue et al. found that proximal gastric cancer patients had lower rates than distal gastric cancer patients in terms of 1-, 3-, and 5-year OS [12]; this may be related to the location of surgical resection and reconstruction method. We found that positive node metastasis, undifferentiated type, and non-*H. pylori* eradication were statistically significantly independent risk factors of postoperative recurrence and death in patients with EGC. However, Choi J et al. found that eradication of *H pylori* after endoscopic resection of gastric tumors did not significantly reduce the incidence of metachronous gastric carcinoma in a prospective trial [13]. While Il Ju Choi et al. found that patients with EGC who received *H. pylori* treatment had lower rates of metachronous GC and more improvement from baseline in the grade of gastric corpus atrophy than patients who received placebo [14]. As for why there is such a difference, we have some speculations, firstly, the gut microbiota after gastrectomy showed higher species diversity and richness, together with greater abundance of aerobes, facultative anaerobes, and oral microbes [15]. Secondly, recent study showed that the diversity of gastric microbiota is lower in *H. pylori*-infected individuals than in non-H. pylori-infected individuals [16]. The diversity of gastric microbiota could be restored to a level similar to that in non- *H. pylori*-infected individuals after successful *H. pylori* eradication [17]. We speculate that the differences in gut microbiota between different populations may lead to different effects of *H. pylori* eradication on the prognosis of patients with EGC.

Our study has several limitations. Firstly, total case number may not be sufficient. Secondly, all of the patients included were from Hunan Province and may not be applicable to populations in other regions. Thirdly, this is a retrospective study, whose level of evidence is lower than that of randomized controlled trials, which may affect the reliability of the conclusions of this study.

## Conclusion

In this study, *H. pylori* eradication resulted in a reduced risk of recurrence in patients with EGC after radical gastrectomy and a better prognosis than in those without *H. pylori* eradication. This study provides evidence-based information on the prophylactic effect of *H. pylori* eradication on the postoperative recurrence of EGC, which may serve as a reference for determining whether or not to eradicate *H. pylori* in patients with EGC after radical gastrectomy. In conclusion, *H. pylori* eradication treatment could prevent the recurrence of postoperative EGC to prolong the overall survival of patients with EGC.

## Data Availability

Not applicable.
